# Comparison of the effects of recombinant human soluble thrombomodulin for systemic inflammatory response syndrome-associated coagulopathy with and without continuous hemodiafiltration

**DOI:** 10.1186/cc9703

**Published:** 2011-03-11

**Authors:** SM Matsuo, T Ikeda, K Ikeda, H Ikeda, S Suda, M Hiramatsu

**Affiliations:** 1 Tokyo Medical University, Hachioji Medical Center, Tokyo, Japan

## Introduction

Recombinant human soluble thrombomodulin (rhs-TM) has a potent anticoagulant effect on septic disseminated intravascular coagulation (DIC) by binding to thrombin and activating protein C. The infusion dosage of rhs-TM should be reduced for patients with renal failure. The aim of this study was to compare the effects of rhs-TM for systemic inflammatory response syndrome (SIRS)-associated coagulopathy (SAC) with and without continuous hemodiafiltration (CHDF).

## Methods

The subjects were 12 patients with SAC treated with rhs-TM in our ICU. Of these, six received 380 units/kg/day rhs-TM, and six who were undergoing CHDF received 130 units/kg/day for 6 to 7 days. We analyzed the changes in DIC, sequential organ failure assessment (SOFA) and SIRS scores, platelet counts, antithrombin levels, fibrin/fibrinogen degradation products (FDP) and prothrombin time internationalized ratio (PT-INR) after each treatment with rhs-TM. The values are expressed as means ± SD and were analyzed using Student's paired *t *test and the Wilcoxon *t *test (*P *< 0.05).

## Results

SOFA, DIC and SIRS scores and the values of PT-INR decreased after the administration of rhs-TM in both groups. Platelet counts increased in the group without CHDF and decreased in the group with CHDF, but these changes were not statistically significant. Antithrombin levels also increased in both groups, but these changes were not statistically significant either. FDP decreased significantly only in the group without CHDF. The changes in platelet counts were influenced by CHDF, because platelet counts were decreased only in the group with CHDF. Several reports have mentioned that rhs-TM has an effect of decreasing FDP for SAC. In this study, we observed decreased FDP only in the group without CHDF. We speculate that these results were influenced by an infusion dose of rhs-TM.

## Conclusions

rhs-TM has a potent effect in improving septic DIC even with an infusion dose of 130 units/kg/day for patients with CHDF.

